# Race, Ethnicity, Language, Social Class, and Health Communication Inequalities: A Nationally-Representative Cross-Sectional Study

**DOI:** 10.1371/journal.pone.0014550

**Published:** 2011-01-18

**Authors:** Kasisomayajula Viswanath, Leland K. Ackerson

**Affiliations:** 1 Center for Community-Based Research, Dana-Farber Cancer Institute, Boston, Massachusetts, United States of America; 2 Department of Society, Human Development, and Health, Harvard School of Public Health, Boston, Massachusetts, United States of America; 3 Department of Community Health and Sustainability, University of Massachusetts Lowell, Lowell, Massachusetts, United States of America; Swiss Paraplegic Research, Switzerland

## Abstract

**Background:**

While mass media communications can be an important source of health information, there are substantial social disparities in health knowledge that may be related to media use. The purpose of this study is to investigate how the use of cancer-related health communications is patterned by race, ethnicity, language, and social class.

**Methodology/Principal Findings:**

In a nationally-representative cross-sectional telephone survey, 5,187 U.S. adults provided information about demographic characteristics, cancer information seeking, and attention to and trust in health information from television, radio, newspaper, magazines, and the Internet. Cancer information seeking was lowest among Spanish-speaking Hispanics (odds ratio: 0.42; 95% confidence interval: 0.28–0.63) compared to non-Hispanic whites. Spanish-speaking Hispanics were more likely than non-Hispanic whites to pay attention to (odds ratio: 3.10; 95% confidence interval: 2.07–4.66) and trust (odds ratio: 2.61; 95% confidence interval: 1.53–4.47) health messages from the radio. Non-Hispanic blacks were more likely than non-Hispanic whites to pay attention to (odds ratio: 2.39; 95% confidence interval: 1.88–3.04) and trust (odds ratio: 2.16; 95% confidence interval: 1.61–2.90) health messages on television. Those who were college graduates tended to pay more attention to health information from newspapers (odds ratio: 1.98; 95% confidence interval: 1.42–2.75), magazines (odds ratio: 1.86; 95% confidence interval: 1.32–2.60), and the Internet (odds ratio: 4.74; 95% confidence interval: 2.70–8.31) and had less trust in cancer-related health information from television (odds ratio: 0.44; 95% confidence interval: 0.32–0.62) and radio (odds ratio: 0.54; 95% confidence interval: 0.34–0.86) compared to those who were not high school graduates.

**Conclusions/Significance:**

Health media use is patterned by race, ethnicity, language and social class. Providing greater access to and enhancing the quality of health media by taking into account factors associated with social determinants may contribute to addressing social disparities in health.

## Introduction

The role of mass media and interpersonal communication in influencing health is widely acknowledged.[1], [2], [3] The function that communication plays in influencing health spans the entire disease continuum including prevention, diagnosis, treatment, survivorship, and end-of life care.[4], [5] Information and knowledge about health has been shown to benefit those who use it in preventing, getting treatment for, recovering from, and dealing with the physical and psychological consequences of illness.[6], [7], [8], [9]

Recent studies have shown that the benefits of health information are not equally distributed across socially distinct groups in the United States, and in fact there appear to be disparities in how people attend to and take advantage of health information.[10], [11] These inequalities in communication parallel with disparities in health. Communication inequality has been offered as one potential mechanism linking social determinants to health outcomes in the structural information model (SIM).[8] In brief, the SIM suggests that outcomes in individual and population health could be understood by examining how social determinants such as race, ethnicity, and class are related to how people access, seek, process, and use health information.

Three key dimensions of communication include health information seeking, attention to health in the media, and trust in the media. *Health information seeking* is a measure of how actively people look for health information.[12] This is a highly valued skill in the current consumer-driven health approach where people are expected to participate in decisions about their health, and information is a necessary resource in making those decisions.[13], [14], [15], [16], [17] While it is commonly assumed that those affected by major diseases actively seek information, there is potential for disparities in information seeking among people of different socioeconomic position.[18] *Attention to health information* is an indicator of degree of interest in a topic and is related to health knowledge.[3] *Trust* is an indicator of esteem with which people hold a source of information and the extent to which they view the information believable.[19] While the physician has traditionally been a highly trusted information source, the emergence of digital media makes it important to document the patterns of trust in various information sources and the relation to social determinants in this new era.[20]

The purpose of this study is to use a nationally-representative sample to investigate the relationships that race, ethnicity, language, and social class have with the use of health communications including cancer information seeking, attention to health information in the mass media, and trust of cancer information from these media.

## Methods

### Data

The data for this project came from the 2003 Health Information National Trends Survey (HINTS 2003), a nationally-representative dataset of 6,369 US adults.[21] Briefly, HINTS is an initiative of the National Cancer Institute to collect national data about health communications, cancer knowledge and beliefs, and cancer-related behaviors. In the survey, US residents, regardless of citizenship, were selected through random digit dialing with oversampling of Hispanics and non-Hispanic blacks. Between October 2002 and April 2003, trained interviewers conducted computer-assisted telephone interviews in either English or Spanish, according to the preferred language of the respondent. The response rates were 55 percent for the household screener and 62.8 percent for the extended interview.

**Figure 1:**
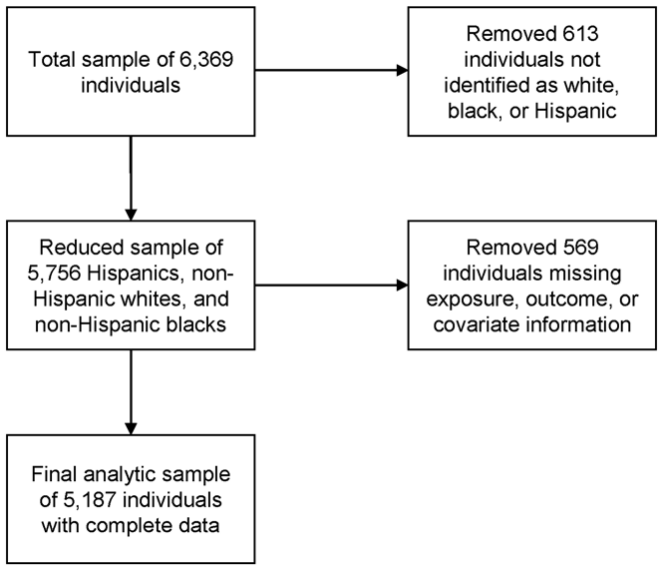
Selection of eligible participants from the 2003 Health Information National Trends Survey.

Since racial and ethnic identity was an important aspect of this study, we limited the dataset to those individuals who provided information about their racial and ethnic heritage. We also made an *a priori* decision to remove from the analysis those individuals from ethnic groups for whom our sample had an inadequate number of individuals to properly analyze. As a result, we removed 613 people of various racial and ethnic groups and limited our analysis to Hispanics, non-Hispanic blacks, and non-Hispanic whites where we had sufficient numbers (Figure 1). An additional 569 subjects were removed from the analyses due to missing information about the outcomes, predictors, or important covariates for a final sample size of 5,187.

### Outcome measures

This study investigated three main outcomes: cancer information seeking, attention to health information sources, and trust in cancer information sources. We measured cancer information seeking with the item, “Have you ever looked for information about cancer from any source?” with the answer given as yes or no. Attention was measured for five different health information sources: television, radio, Internet, newspaper, and magazines. Attention was measured using the question, “How much attention do you pay to information about health or medical topics [from this source]?” Trust was measured for each of seven cancer information sources: doctors or other health professionals, family or friends, television, radio, Internet, newspapers, and magazines. Trust was measured using the question, “How much would you trust the information about cancer from [this source]?” Answers for the trust and attention questions were a lot, some, a little, or not at all. We gave careful consideration to the use of these attention and trust variables in the analyses with the intention of dichotomizing the responses into high and low groups. Previous research has shown that positive changes in cancer knowledge and beliefs occur at the highest levels of trust and attention to health messages.[11] We conducted preliminary analyses dichotomizing the responses to these variables as “a lot or some” and “a little or not at all” trust or attention. These analyses showed that, under these circumstances, for nearly all of the variables, the response category “a lot or some” composed over half of the responses, indicating that it was a normative behavior (Table S1). As we were concerned with modeling non-normative levels of high trust and attention, we made a deliberate decision to collapse the responses to all of the trust and attention questions to create binary responses of either a lot or not a lot (some, a little, or not at all).

### Independent Variables

Self-reported race and ethnicity variables were combined into a single race/ethnicity variable. Interviewees selected whether they wanted to be interviewed in English or Spanish depending on which language they were more comfortable speaking. Preliminary investigations indicated that the Hispanic participants in the sample were evenly split between those preferring English and Spanish. In consideration of previous research finding variations in mass media use among Hispanics according to language,[22] the race/ethnicity variable was expanded into a race/ethnicity/language variable with the categories non-Hispanic white, non-Hispanic black, English-speaking Hispanic, and Spanish-speaking Hispanic. Education was categorized according to the highest level achieved as less than high school, high school graduate, some college, or college graduate. Annual household income was measured with the categories less than $25,000; $25,000–$34,999; $35,000–$49,999; $50,000–$74,999; or $75,000 or more.

### Covariates

A number of covariates that were hypothesized to be associated with both sociodemographic characteristics and health media use were included in the models namely sex, age, marital status, employment, rural/urban residence, health insurance, presence of children under age 18 in the household, having had cancer, and having had a family member with cancer. Gender was a binary variable recorded as male or female. Age was grouped in the following categories: 18–34; 35–49; 50–64; 65–74; or 75 and older. Marital status was divided into two groups: those who were married or cohabiting and those who were not married or cohabiting. Employment was measured as employed, homemaker, student, retired, or not employed. Rural/urban residence was determined from county-level reports of the Economic Research Service of the United States Department of Agriculture [23] and was grouped in the following categories: counties in metropolitan areas with one million residents or more, counties in metropolitan areas with less than one million residents, counties in rural areas with 20,000 residents or more, or counties in rural areas with less than 20,000 residents. Health insurance, children under age 18, history of cancer, and family history of cancer were each coded as yes or no.

### Data analysis

Each outcome was modeled with logistic regression using the proc multilog procedure in SAS-callable SUDAAN version 9.0. Survey weights were applied to each model to account for multiple adjustments in the sampling procedure to ensure that the sample was representative of all US adults. A jackknife method was used to calculate standard errors of parameter estimates.

### Ethical considerations

The data collection procedures, in which subjects provided verbal informed consent, were approved by the National Institutes of Health Office of Human Subjects Research.[21] The use of this data for the current analysis was approved by the National Institutes of Health, the Harvard School of Public Health Office of Human Research Administration, and the University of Massachusetts Lowell Institutional Review Board.

## Results

### Descriptive statistics

Over three quarters of the sample were non-Hispanic white, 11% were non-Hispanic black, with the remaining 12% split evenly between English- and Spanish-speaking Hispanics. Approximately one third of respondents had a high school education with 27% having some college and 25% having a college degree (Table 1). Those from households making less than $25,000 per year comprised 29% of the total, with the remainder split roughly evenly among the four remaining groups. Roughly half of those surveyed reported having sought cancer information previously (Table 2).

**Table 1:** Frequency and weighted percentages of socioeconomic and demographic variables among individuals in the HINTS 2003 survey.

VARIABLE	N	Weighted %
Race/ethnicity		
Non-Hispanic White	3845	76.8
English-speaking Hispanic	399	6.4
Spanish-speaking Hispanic	299	6.0
Non-Hispanic Black	644	10.7
Education		
Less than high school	602	15.8
High school	1554	32.1
Some college	1406	27.5
College graduate	1625	24.6
Household income		
<$25,000	1561	28.6
$25,000 to <$35,000	744	13.7
$35,000 to <$50, 000	892	17.3
$50,000 to <$75,000	876	17.5
≥$75,000	1114	22.8
Employment		
Employed	3161	61.9
Homemaker	420	7.9
Student	199	5.7
Retired	898	14.5
Not employed	509	10.0
Urbanicity		
Metro region ≥1 million	2539	48.5
Metro region <1 million	1666	32.4
Rural region ≥20,000	391	7.4
Rural region <20,000	591	11.7
Age (in years)		
18–34	1344	30.0
35–49	1657	32.5
50–64	1235	22.1
65–74	541	9.3
75+	410	6.1
Gender		
Male	2071	48.3
Female	3116	51.7
Marital status		
Married or committed	2966	65.1
Not married	2221	34.9
Health insurance		
Yes	4533	85.7
No	654	14.3
Children under age 18		
No	3146	57.0
Yes	2041	43.0
History of cancer		
No	4566	89.2
Yes	621	10.9
Family history of cancer		
No	1890	36.8
Yes	3297	63.2
**TOTAL**	**5187**	**100.0**

**Table 2:** Frequency and weighted percentages of health media use variables among individuals in the HINTS 2003 survey.

VARIABLE	N	Weighted %
Information seeking		
Yes	2502	46.1
No	2685	53.9
Attend television		
A lot	1788	32.3
Not a lot	3399	67.7
Attend radio		
A lot	828	15.1
Not a lot	4359	84.9
Attend newspaper		
A lot	1321	24.7
Not a lot	3866	75.3
Attend magazines		
A lot	1341	24.2
Not a lot	3846	75.8
Attend Internet		
A lot	690	12.7
Not a lot	4497	87.3
Trust doctors		
A lot	3201	61.7
Not a lot	1986	38.3
Trust family and friends		
A lot	940	18.5
Not a lot	4247	81.5
Trust newspaper		
A lot	644	12.7
Not a lot	4543	87.3
Trust magazines		
A lot	807	15.7
Not a lot	4380	84.3
Trust radio		
A lot	466	9.7
Not a lot	4721	90.4
Trust Internet		
A lot	1222	23.9
Not a lot	3965	76.1
Trust television		
A lot	988	19.6
Not a lot	4199	80.4
**TOTAL**	**5187**	**100.0**

A sensitivity analysis investigated the characteristics of individuals who were excluded from the main analyses because they provided incomplete data revealed evidence of disproportionally missing values (Table S2). Notably, those individuals who were most likely to be missing information were Spanish-speaking Hispanics, females, people aged 65 and older, and people who had less than a high school education.

### Cancer information seeking

Data in Table 3 indicate that, compared to non-Hispanic whites, Spanish-speaking Hispanics (odds ratio [OR] = 0.42; 95% confidence interval [CI] = 0.28–0.63) and English-speaking Hispanics (OR = 0.74; 95% CI = 0.55–1.00) were less likely to seek cancer information. Socioeconomic gradients were apparent such that those with higher levels of education and income were more likely to report seeking cancer information.

**Table 3:** Odd ratios (OR) and 95% Confidence Intervals (CI) of the association of cancer information seeking and paying a lot of attention to health media with race/ethnicity/language and social characteristics among U.S. adults in the HINTS 2003 study.

	Information Seeking		Attend Television		Attend Radio	
	OR	95% CI	OR	95% CI	OR	95% CI
Ethnicity						
Non-Hispanic White (ref)	1.00		1.00		1.00	
English-speaking Hispanic	0.74	(0.55, 1.00)	1.12	(0.86, 1.47)	1.15	(0.76, 1.74)
Spanish-speaking Hispanic	0.42	(0.28, 0.63)	2.59	(1.82, 3.69)	3.10	(2.07, 4.66)
Non-Hispanic Black	0.90	(0.66, 1.21)	2.39	(1.88, 3.04)	1.98	(1.46, 2.68)
Education						
Less than high school (ref)	1.00		1.00		1.00	
High school	1.13	(0.85, 1.51)	1.12	(0.85, 1.49)	1.11	(0.77, 1.60)
Some college	1.66	(1.22, 2.27)	1.16	(0.86, 1.58)	1.20	(0.79, 1.81)
College graduate	2.74	(1.98, 3.81)	1.25	(0.93, 1.70)	1.39	(0.96, 2.01)
Annual household income						
<$25,000 (ref)	1.00		1.00		1.00	
$25,000 to <$35,000	1.31	(0.97, 1.77)	1.09	(0.88, 1.34)	0.91	(0.66, 1.25)
$35,000 to <$50,000	1.09	(0.86, 1.38)	0.90	(0.73, 1.10)	0.78	(0.52, 1.17)
$50,000 to <$75,000	1.53	(1.17, 2.00)	1.08	(0.81, 1.43)	1.05	(0.73, 1.51)
> = $75,000	1.56	(1.14, 2.15)	1.01	(0.78, 1.30)	0.92	(0.63, 1.36)

	Attend Newspaper		Attend Magazines		Attend Internet	
	OR	95% CI	OR	95% CI	OR	95% CI
Ethnicity						
Non-Hispanic White (ref)	1.00		1.00		1.00	
English-speaking Hispanic	0.92	(0.63, 1.33)	0.87	(0.62, 1.22)	1.00	(0.64, 1.58)
Spanish-speaking Hispanic	1.25	(0.76, 2.05)	1.67	(1.04, 2.66)	0.53	(0.25, 1.13)
Non-Hispanic Black	1.65	(1.25, 2.19)	1.87	(1.48, 2.36)	1.26	(0.85, 1.87)
Education						
Less than high school (ref)	1.00		1.00		1.00	
High school	1.44	(1.09, 1.90)	1.35	(1.00, 1.83)	2.04	(1.13, 3.68)
Some college	1.86	(1.33, 2.62)	1.90	(1.38, 2.61)	3.80	(2.02, 7.15)
College graduate	1.98	(1.42, 2.75)	1.86	(1.32, 2.60)	4.74	(2.70, 8.31)
Annual household income						
<$25,000 (ref)	1.00		1.00		1.00	
$25,000 to <$35,000	0.90	(0.69, 1.18)	0.93	(0.68, 1.26)	0.96	(0.53, 1.75)
$35,000 to <$50,000	0.76	(0.55, 1.05)	0.89	(0.63, 1.26)	1.04	(0.56, 1.92)
$50,000 to <$75,000	0.88	(0.61, 1.26)	1.20	(0.84, 1.73)	1.18	(0.61, 2.31)
> = $75,000	0.84	(0.62, 1.15)	1.06	(0.75, 1.50)	1.24	(0.62, 2.46)

Note: All models are additionally adjusted for employment, marital status, age, gender, rural/urban residence, health insurance coverage, children living in the home, personal cancer diagnosis, and family cancer diagnosis.

### Attention to health information sources

Non-Hispanic blacks were more likely to pay attention to health messages on television (OR = 2.39; 95% CI = 1.88–3.04), on the radio (OR = 1.98; 95% CI = 1.46–2.68), in newspapers (OR = 1.65; 95% CI = 1.25–2.19), and in magazines (OR = 1.87; 95% CI = 1.48–2.36) compared to non-Hispanic whites (Table 3). At the same time, Spanish-speaking Hispanics were more likely to pay attention to health messages on television (OR = 2.59; 95% CI = 1.82–3.69), on the radio (OR = 3.10; 95% CI = 2.07–4.66), and in magazines (OR = 1.67; 95% CI = 1.04–2.66) compared to non-Hispanic whites. Higher levels of education were associated with higher likelihood of paying a lot of attention to health information in various mass media sources. Household income had no association with attending to health messages.

### Trust in cancer information from media sources

Non-Hispanic blacks reported increased trust in cancer information from television (OR = 2.16; 95% CI = 1.61–2.90), radio (OR = 1.89; 95% CI = 1.20–2.98), newspapers (OR = 1.59; 95% CI = 1.10–2.31), and magazines (OR = 2.05; 95% CI = 1.46–2.88) compared to whites (Table 4). English-speaking Hispanics were more likely to report a lot of trust in cancer information from television (OR = 1.34; 95% CI = 1.01–1.79), and Spanish-speaking Hispanics were more likely to report a lot of trust in cancer information from both television (OR = 1.74; 95% CI = 1.14–2.65) and radio (OR = 2.61; 95% CI = 1.53–4.47) compared to non-Hispanic whites. College graduates were significantly less likely to report a lot of trust in cancer information from television and radio than those with less than high school education. An increased likelihood of reporting a lot of trust in cancer information from the Internet was found among those from upper income households.

**Table 4:** Odd ratios (OR) and 95% Confidence Intervals (CI) of the association of having a lot of trust in cancer information from media and interpersonal sources with race/ethnicity/language and social class characteristics among U.S. adults in the HINTS 2003 study.

	Trust Television		Trust Radio		Trust Newspaper		Trust Magazines	
	OR	95% CI	OR	95% CI	OR	95% CI	OR	95% CI
Ethnicity								
Non-Hispanic White (ref)	1.00		1.00		1.00		1.00	
English-speaking Hispanic	1.34	(1.01, 1.79)	1.27	(0.81, 1.98)	1.23	(0.80, 1.88)	1.28	(0.86, 1.89)
Spanish-speaking Hispanic	1.74	(1.14, 2.65)	2.61	(1.53, 4.47)	1.66	(0.86, 3.21)	1.37	(0.78, 2.41)
Non-Hispanic Black	2.16	(1.61, 2.90)	1.89	(1.20, 2.98)	1.59	(1.10, 2.31)	2.05	(1.46, 2.88)
Education								
Less than high school (ref)	1.00		1.00		1.00		1.00	
High school	0.78	(0.58, 1.05)	0.86	(0.61, 1.22)	1.22	(0.87, 1.71)	1.05	(0.73, 1.50)
Some college	0.72	(0.50, 1.02)	0.60	(0.32, 1.12)	1.15	(0.72, 1.83)	1.08	(0.67, 1.75)
College graduate	0.44	(0.32, 0.62)	0.54	(0.34, 0.86)	1.05	(0.67, 1.65)	1.10	(0.70, 1.72)
Annual household income								
<$25,000 (ref)	1.00		1.00		1.00		1.00	
$25,000 to <$35,000	0.84	(0.59, 1.19)	0.70	(0.43, 1.15)	0.71	(0.49, 1.03)	0.84	(0.59, 1.21)
$35,000 to <$50,000	0.72	(0.47, 1.12)	0.72	(0.41, 1.26)	0.64	(0.40, 1.04)	0.69	(0.44, 1.09)
$50,000 to <$75,000	0.87	(0.57, 1.32)	0.81	(0.44, 1.50)	0.69	(0.42, 1.12)	0.84	(0.53, 1.32)
> = $75,000	0.75	(0.48, 1.17)	0.66	(0.34, 1.28)	0.68	(0.43, 1.09)	0.67	(0.43, 1.04)

	Trust Internet		Trust Doctor		Trust Family and Friends			
	OR	95% CI	OR	95% CI	OR	95% CI		
Ethnicity								
Non-Hispanic White (ref)	1.00		1.00		1.00			
English-speaking Hispanic	1.03	(0.76, 1.40)	0.84	(0.63, 1.11)	0.89	(0.59, 1.34)		
Spanish-speaking Hispanic	0.97	(0.57, 1.64)	1.36	(0.92, 2.01)	1.33	(0.88, 2.00)		
Non-Hispanic Black	1.28	(0.96, 1.70)	0.89	(0.71, 1.12)	1.29	(0.98, 1.70)		
Education								
Less than high school (ref)	1.00		1.00		1.00			
High school	1.05	(0.70, 1.57)	1.24	(0.98, 1.57)	1.02	(0.74, 1.42)		
Some college	0.93	(0.61, 1.43)	1.17	(0.91, 1.50)	0.95	(0.66, 1.37)		
College graduate	1.08	(0.69, 1.68)	1.71	(1.35, 2.16)	0.83	(0.59, 1.18)		
Annual household income								
<$25,000 (ref)	1.00		1.00		1.00			
$25,000 to <$35,000	1.38	(1.03, 1.86)	0.88	(0.71, 1.08)	0.79	(0.62, 1.02)		
$35,000 to <$50,000	1.07	(0.77, 1.47)	0.95	(0.74, 1.22)	0.71	(0.50, 1.01)		
$50,000 to <$75,000	1.52	(1.13, 2.04)	1.02	(0.81, 1.29)	0.56	(0.39, 0.80)		
> = $75,000	1.34	(1.00, 1.81)	1.11	(0.85, 1.46)	0.54	(0.39, 0.75)		

Note: All models are additionally adjusted for employment, marital status, age, gender, rural/urban residence, health insurance coverage, children living in the home, personal cancer diagnosis, and family cancer diagnosis.

### Trust in cancer information from interpersonal channels

Ethnicity was unrelated to trust in cancer information from interpersonal channels (Table 4). However, education was directly associated with reporting a lot of trust in cancer information from doctors and other medical professionals and income was inversely associated with reporting a lot of trust in cancer information from family and friends.

## Discussion

Race and ethnicity are social characteristics that, in an immigrant population in the United States, are often linked with the issue of English language proficiency. Our results indicated that the combination of race and ethnicity with language strongly influence health communication behaviors. The inability to speak fluent English may hinder the search for cancer information among Spanish-speaking Hispanics by limiting the available information sources and health content.[24] That both English- and Spanish-speaking Hispanics have lower rates of cancer information seeking may also result from culturally-based concepts of fatalism [25] and deference to medical professionals regarding health decisions.[26] Conversely, Spanish-speaking Hispanics and non-Hispanic blacks are the ethnic groups that are most likely to pay a lot of attention to and have a lot of trust in cancer messages from all kinds of media, excluding the Internet. Increased trust in cancer information from these media sources may result from social capital [27] that comes from the reliance of these populations on ethnically-targeted media outlets.[28], [29]

We found education to be a consistently strong determinant of health communication behaviors. With the bewildering array of choices available in the information environment,[30] education may provide consumers with the skills, knowledge, and confidence to seek specific health information of interest, accounting for the educational gradient in cancer information seeking. The educational gradients in attention to health information from newspapers, magazines, and the Internet may arise from the fact that interpretation of information from these sources requires relatively high levels of literacy,[31], [32] particularly when compared to information from television and radio. Additionally, education may enable people to communicate more effectively with medical professionals making them more trusting of information from this source.[33] An extensive review has shown that social class influences physician-patient interaction with social advantages potentially leading to better health outcomes.[34] At the same time, education may allow individuals to sift through conflicting information in the media environment with a critical eye to determine what information is most relevant and truthful.[18] Since the overwhelming majority of television and radio programming in the United States is supported by commercial interests that sometimes conflict with health promoting messages, those with higher levels of education may tend to be less trusting of health information from television and radio.

While higher incomes could provide the resources to purchase the means to access the Internet such as broadband connections and computer hardware,[35] it is also likely that higher income individuals more frequently use computers as part of their employment.[36] This increased access may in turn promote familiarity with and trust in the Internet that provides an individual with confidence and technical knowledge necessary to seek information about health. Access to numerous trusted sources of health information may also account for reduced reliance on information from other informal health sources such as friends and family.

Our data also go beyond the widely documented “digital divide” in which those of lower socioeconomic status have less access to the Internet [37], [38] by examining a gamut of information services available to a consumer today. Our research indicates that, apart from simply accessing the Internet, individuals of lower educational status are less likely to pay a lot of attention to and less likely to have a lot of trust in health information on the Internet. It is possible that lack of familiarity with the medium and the complexity in navigating the Web could potentially deter people from a lower socioeconomic status to make effective use of the Internet for health.

We document differences across a variety of media regarding how familiarity and use of the media may influence information seeking, attention, and trust. This information suggests a number of ways that mass media can be used to promote health among particular populations. For example, the evidence indicates that health and medical professionals might effectively use television and radio to communicate health preserving messages to non-Hispanic blacks and Spanish-speaking Hispanics given their affinity for these media. Institutions that use media to communicate health information could make efforts to construct messages in formats and manners that are accessible to a variety of groups. Additionally, projects that promote access to broadband services and training in the use of the Internet, particularly among low-income individuals, may promote familiarity with this medium that could allow these individuals to access a wider array of health-promoting information.

### Limitations

While major topics of this study included attention to and trust in cancer information in the mass media, no attempt was made to measure the content of these media sources. For example, health information on television could be interpreted equally as a health theme in a situation comedy, a brief story on the evening news, or a thorough and balanced documentary. Since a majority of mass media dissemination in the United States is supported by commercial interests, particularly in the fields of television and radio, it is likely that individuals who reported using these media were exposed to substantial amounts of paid advertising. This study did not distinguish between health media messages that were intended to be informational and those that were intended to sell health-influencing products. Furthermore, the survey data did not distinguish between those media outlets intended for a general audience and ethnic media meant to appeal to a specific cultural group. Thus, it may be that people of different ethnicities tend to conceive of health media in different ways in relation to their own social group and thus differentially report trust and attention. Additionally, the measures of information seeking and trust asked the respondents about attitudes towards cancer information in the media, and may not be representative of media-related behaviors that deal with other types of health information. The observational nature of the data prevents us from making causal inferences from our results. However, the outcomes of interest in this study—aspects of health media use—are unlikely to precede the exposures under investigation—race/ethnicity/language, education, and income—thereby reducing the chance of the results reflecting reverse causation.

In terms of sample selection, the use of random digit dialing to recruit participants for this study would tend to exclude those individuals without home telephones and those who spend little time at home. In addition, we removed 569 individuals who were missing information about an outcome, exposure, or covariate of interest. While this number was less than 10% of the original sample, there was some patterning of missing variables. Individuals who were Spanish-speaking Hispanics, female, aged 65 or older, and who never graduated from high school were more likely to be removed from the analysis. Finally, the sample sizes were inadequate to allow for the analysis of races and ethnicities other than Hispanics, non-Hispanic whites, and non-Hispanic blacks so we were forced to remove individuals who did not identify with one of these groups from the analysis. These factors challenge the nationally-representative nature of the final sample analyzed in this study.

### Conclusion

These data show that important social determinants such as race, ethnicity, language, and social class that have been found to influence health outcomes are also strongly linked to health communication behaviors such as cancer information seeking, attention to health in the media, and trust in cancer information from communication sources. The data also point out potential ways to reach the underserved to bridge current disparities in health by improving access to and quality of the health information for socially marginalized groups.

## Supporting Information

Table S1 Frequency of health media use variables dichotomized by combining responses.
(0.05 MB DOC)

Table S2 Association of missing values with outcome and covariate variables.
(0.15 MB DOC)
