# Supplementary material for: Race, Ethnicity, Language, Social Class, and Health Communication Inequalities: A Nationally-Representative Cross-Sectional Study
Source: PLoS One. 2011 Jan 18;6(1):e14550. doi: 10.1371/journal.pone.0014550 (PMC3022647; doi:10.1371/journal.pone.0014550)
Supplement: Table S1 — Frequency of health media use variables dichotomized by combining responses. (0.05 MB DOC) [file pone.0014550.s001.doc]

| VARIABLE | N | | Weighted % | |
| --- | --- | --- | --- | --- |
| Attend television |  |  | |  |
| A lot or some | 3773 | 71.4 | |  |
| A little or not at all | 1414 | 28.6 | |  |
| Attend radio |  |  | |  |
| A lot or some | 2293 | 43.8 | |  |
| A little or not at all | 2894 | 56.2 | |  |
| Attend newspaper |  |  | |  |
| A lot or some | 2980 | 55.8 | |  |
| A little or not at all | 2207 | 44.2 | |  |
| Attend magazines |  |  | |  |
| A lot or some | 2925 | 53.2 | |  |
| A little or not at all | 2262 | 46.8 | |  |
| Attend Internet |  |  | |  |
| A lot or some | 1520 | 28.0 | |  |
| A little or not at all | 3667 | 72.0 | |  |
| Trust television |  |  | |  |
| A lot or some | 3728 | 71.5 | |  |
| A little or not at all | 1459 | 28.5 | |  |
| Trust radio |  |  | |  |
| A lot or some | 2868 | 54.7 | |  |
| A little or not at all | 2319 | 45.3 | |  |
| Trust newspaper |  |  | |  |
| A lot or some | 3360 | 64.1 | |  |
| A little or not at all | 1827 | 35.9 | |  |
| Trust magazines |  |  | |  |
| A lot or some | 3552 | 67.1 | |  |
| A little or not at all | 1635 | 32.9 | |  |
| Trust Internet |  |  | |  |
| A lot or some | 3349 | 64.8 | |  |
| A little or not at all | 1838 | 35.2 | |  |
| Trust doctors |  |  | |  |
| A lot or some | 4820 | 92.2 | |  |
| A little or not at all | 367 | 7.8 | |  |
| Trust family and friends |  |  | |  |
| A lot or some | 3487 | 67.5 | |  |
| A little or not at all | 1700 | 32.5 | |  |
| TOTAL | 5187 | 100.0 | |  |
