# Supplementary material for: Race, Ethnicity, Language, Social Class, and Health Communication Inequalities: A Nationally-Representative Cross-Sectional Study
Source: PLoS One. 2011 Jan 18;6(1):e14550. doi: 10.1371/journal.pone.0014550 (PMC3022647; doi:10.1371/journal.pone.0014550)
Supplement: Table S2 — Association of missing values with outcome and covariate variables. (0.15 MB DOC) [file pone.0014550.s002.doc]

| Variable | Number in analysis | Number excluded from analysis | % excluded from analysis | P-value |
| --- | --- | --- | --- | --- |
| Race/ethnicity/language |  |  |  | 0.0081 |
| Non-Hispanic White | 3845 | 431 | 10.1 |  |
| English-speaking Hispanic | 399 | 31 | 7.2 |  |
| Spanish-speaking Hispanic | 299 | 63 | 17.4 |  |
| Non-Hispanic Black | 644 | 72 | 10.1 |  |
| Education |  |  |  | 0.0032 |
| Less than high school | 602 | 86 | 12.5 |  |
| High school | 1554 | 182 | 10.5 |  |
| Some college | 1406 | 135 | 8.8 |  |
| College graduate | 1625 | 162 | 9.1 |  |
| Household income |  |  |  | 0.7130 |
| < $25,000 | 1561 | 30 | 1.9 |  |
| $25,000 to < $35,000 | 744 | 9 | 1.2 |  |
| $35,000 to < $50, 000 | 892 | 10 | 1.1 |  |
| $50,000 to < $75,000 | 876 | 17 | 1.9 |  |
| ≥ $75,000 | 1114 | 23 | 2.0 |  |
| Employment |  |  |  | 0.0001 |
| Employed | 3161 | 251 | 7.4 |  |
| Homemaker | 420 | 73 | 14.8 |  |
| Student | 199 | 39 | 16.4 |  |
| Retired | 898 | 139 | 13.4 |  |
| Not employed | 509 | 54 | 9.6 |  |
| Urbanicity |  |  |  | 0.3872 |
| Metro region ≥ 1 million | 2539 | 308 | 10.8 |  |
| Metro region < 1 million | 1666 | 183 | 9.9 |  |
| Rural region ≥ 20,000 | 391 | 49 | 11.1 |  |
| Rural region < 20,000 | 591 | 57 | 8.8 |  |
| Age (in years) |  |  |  | 0.0007 |
| 18-34 | 1344 | 133 | 9.0 |  |
| 35-49 | 1657 | 136 | 7.6 |  |
| 50-64 | 1235 | 134 | 9.8 |  |
| 65-74 | 541 | 95 | 14.9 |  |
| 75+ | 410 | 81 | 16.5 |  |
| Gender |  |  |  | 0.0022 |
| Male | 2071 | 188 | 8.3 |  |
| Female | 3116 | 409 | 11.6 |  |
| Marital status |  |  |  | 0.0031 |
| Married or committed | 2966 | 284 | 8.7 |  |
| Not married | 2221 | 275 | 11.0 |  |
| Health insurance |  |  |  | 0.8429 |
| Yes | 4533 | 497 | 9.9 |  |
| No | 654 | 61 | 8.5 |  |
| Children under age 18 |  |  |  | 0.0037 |
| No | 3146 | 408 | 11.5 |  |
| Yes | 2041 | 161 | 7.3 |  |
| History of cancer |  |  |  | 0.6294 |
| No | 4566 | 500 | 9.9 |  |
| Yes | 621 | 82 | 11.7 |  |
| Family history of cancer |  |  |  | 0.0025 |
| No | 1890 | 229 | 10.8 |  |
| Yes | 3297 | 326 | 9.0 |  |
| Information seeking |  |  |  | 0.0029 |
| Yes | 2502 | 237 | 8.7 |  |
| No | 2685 | 351 | 11.6 |  |
| Attend television |  |  |  | 0.5683 |
| A lot | 1788 | 198 | 10.0 |  |
| Not a lot | 3399 | 399 | 10.5 |  |
| Attend radio |  |  |  | 0.5553 |
| A lot | 828 | 92 | 10.0 |  |
| Not a lot | 4359 | 505 | 10.4 |  |
| Attend newspaper |  |  |  | 0.2953 |
| A lot | 1321 | 152 | 10.3 |  |
| Not a lot | 3866 | 445 | 10.3 |  |
| Attend magazines |  |  |  | 0.0235 |
| A lot | 1341 | 136 | 9.2 |  |
| Not a lot | 3846 | 461 | 10.7 |  |
| Attend Internet |  |  |  | 0.0679 |
| A lot | 690 | 55 | 7.4 |  |
| Not a lot | 4497 | 542 | 10.8 |  |
| Trust doctors |  |  |  | 0.9946 |
| A lot | 3201 | 360 | 10.1 |  |
| Not a lot | 1986 | 237 | 10.7 |  |
| Trust family and friends |  |  |  | 0.3301 |
| A lot | 940 | 112 | 10.6 |  |
| Not a lot | 4247 | 485 | 10.2 |  |
| Trust newspaper |  |  |  | 0.5197 |
| A lot | 644 | 68 | 9.6 |  |
| Not a lot | 4543 | 529 | 10.4 |  |
| Trust magazines |  |  |  | 0.9142 |
| A lot | 807 | 79 | 8.9 |  |
| Not a lot | 4380 | 518 | 10.6 |  |
| Trust radio |  |  |  | 0.4126 |
| A lot | 466 | 52 | 10.0 |  |
| Not a lot | 4721 | 543 | 10.3 |  |
| Trust Internet |  |  |  | 0.1021 |
| A lot | 1222 | 103 | 7.8 |  |
| Not a lot | 3965 | 494 | 11.1 |  |
| Trust television |  |  |  | 0.597 |
| A lot | 988 | 110 | 10.0 |  |
| Not a lot | 4199 | 487 | 10.4 |  |
